# Automated Acoustic Monitoring of Oestrus in Dairy Cows and Sows: Behavioural Evidence, Signal Processing, and Detection Methods

**DOI:** 10.3390/ani16203277

**Published:** 2026-10-17

**Authors:** Jun Wang, Zeping Song, Hui Wang, Shanshan Yin, Kang Hai, Jianghui Liu, Waleid Mohamed EL-Sayed Shakweer

**Affiliations:** 1 School of Information Engineering, Henan University of Science and Technology, Luoyang 471003, China; pzza01@163.com (Z.S.); yss1041529800@126.com (S.Y.); haikang_haust@163.com (K.H.); hl1211_hui@163.com (J.L.); 2 State Key Laboratory of Animal Nutrition, Institute of Animal Science, Chinese Academy of Agricultural Sciences, Beijing 100193, China; wanghui10@caas.cn; 3 Agricultural and Biological Research Institute, National Research Centre, Cairo 12622, Egypt; wm.shakweer@nrc.sci.eg

**Keywords:** automated monitoring, artificial intelligence, bioacoustics, oestrus detection, precision livestock farming, sensor fusion

## Abstract

Oestrus detection determines insemination timing and reproductive efficiency in dairy cow and sow herds, yet manual observation and single-sensor systems remain vulnerable to weak behavioural expression and commercial barn conditions. This structured narrative review organises evidence across biological plausibility, acoustic acquisition, barn interference, feature representation, recognition models, multimodal fusion, decision support, and on-farm validation. Searches of PubMed and Crossref through 3 October 2026 produced 373 records and 339 unique records after deduplication. Of the core database records, 16 met the direct relevance criteria, and 78 supplementary biological, engineering, and methodological sources were incorporated through backward citation searching and targeted contextual retrieval, yielding 94 cited sources. Dairy cow evidence most consistently supports increased vocalisation rate around the oestrus climax and individual acoustic monitoring under defined conditions. Sow evidence depends more strongly on boar stimulation, the standing reflex, and multimodal labels, positioning acoustics mainly as an auxiliary behavioural cue. Reported direct recognition accuracies range from 91.25% to 98.92%, but heterogeneous labels and predominantly internal validation prevent statistical pooling or claims of commercial generalisability. Acoustic monitoring is best positioned as an early-warning and verification layer rather than a standalone predictor of ovulation or optimal insemination timing. Priority needs are standardised reference labels, leakage-resistant animal- and farm-level validation, device robustness, multimodal uncertainty handling, open benchmarks, and evaluation of reproductive and economic outcomes.

## Simple Summary

Detecting when dairy cows and sows are ready for breeding is important for farm efficiency, but visual checks require time and can miss short or night-time events. Animals often change how frequently and how strongly they call around this period. Microphones could therefore provide a continuous and contact-free warning. This review compares the evidence for using animal calls to support automated breeding management in dairy cows and sows. Dairy cow studies most consistently report a rise in calling around the peak of heat, whereas sow calls are more dependent on contact with a boar and confirmation of the standing response. Barn noise, overlapping calls, and difficulty identifying the calling animal still limit practical use. Sound monitoring should therefore complement, rather than replace, activity sensors, cameras, physical checks, and breeding records. Future systems need testing on several farms and must show that they improve breeding decisions and farm outcomes.

## 1. Introduction

In large-scale livestock production, reproductive efficiency directly affects farm profitability, animal utilisation, and management costs. Oestrus detection determines the timing of mating or artificial insemination. Delayed or inaccurate detection can miss the appropriate insemination window, prolonging days open or non-productive days [1]. In dairy cows, conception rate, days open, and productive lifespan are closely linked to economic performance [2]. In sow herds, non-productive days, farrowing rate, and batch reproductive efficiency likewise affect profitability [3,4]. Improving the timeliness and reliability of oestrus detection is therefore a shared priority in intensive dairy cow and sow farming.

Dairy cows and sows represent two contrasting but informative scenarios for precision reproductive monitoring. The comparison is not intended to imply equal maturity of evidence in the two species; instead, dairy cows and sows are used here as contrasting cases for individual-level and pen-level acoustic reproductive monitoring. In modern intensive farming, both dairy cow production and sow production rely heavily on artificial insemination to improve reproductive efficiency and genetic progress [5]. Because insemination requires accurate timing, oestrus detection remains a critical bottleneck in both systems. Biologically, both species show measurable vocal changes during the peri-oestrous period, including changes in vocalisation rate, intensity, duration, and spectral structure [6]. These cues may therefore act as behavioural indicators rather than direct hormonal readouts. The practical deployment routes differ. The high individual value of dairy cows supports the use of neck-mounted sensors, near-field acoustic tags, and activity or location data for individualised monitoring. Sow herd management is organised more strongly around batches and pens, while wearables are vulnerable to biting, impacts, and cleaning [7]. Fixed, centralised, or array-based systems may therefore be more practical in sow facilities [8].

Traditional oestrus detection relies heavily on observing behavioural changes. In dairy cows, relevant signs include mounting, standing to be mounted, and increased activity [9,10]. In sows, detection commonly uses boar stimulation, the back-pressure test, and the standing reflex [11]. Under high-density commercial conditions, manual observation is constrained by labour availability, observer expertise, and challenging barn environments, increasing the likelihood that brief or nocturnal events are overlooked [12]. Precision livestock farming technologies have therefore been adopted to reduce reliance on manual observation. Early automated systems used pedometers and accelerometers to monitor activity and behaviour, but their performance varied among animals and management settings [13,14]. Subsequent studies compared activity, rumination, and location data in dairy cows, and later extended automated detection to beef cattle, sows, and multimodal systems [15,16,17,18]. Across systems, deployment, attachment integrity, and farm routines remain important determinants of reliability [19,20,21].

Existing automated methods have important but distinct limitations. Activity monitors may miss silent heats, weak behavioural expression, or animals housed under movement restrictions [22,23]. Computer vision can capture posture and mounting but remains vulnerable to occlusion, variable lighting, contamination, and high stocking densities [24]. Physiological measurements may align more closely with oestrus status, but cost, sampling complexity, and hygiene requirements limit routine deployment [16]. Moreover, many systems address only binary oestrus classification rather than oestrus stage, ovulation timing, or the optimal insemination window. Acoustic monitoring has attracted interest because vocalisations can be recorded remotely and continuously without directly disturbing animals [25,26,27]. In this context, acoustics is best considered an additional behavioural evidence layer for early warning, behavioural verification, and multimodal decision support.

Previous reviews have examined automated oestrus detection in dairy cows, sow monitoring technologies, livestock bioacoustics, and artificial intelligence in precision livestock farming as largely separate topics [22,28,29,30]. Fewer reviews have evaluated the full evidence chain for acoustic oestrus detection, from biological plausibility to on-farm deployment, across contrasting livestock systems [17]. Accordingly, this review provides an explicit cross-species framework and a practical signpost to the evidence; it is not intended as a systematic review, formal risk-of-bias assessment, or definitive meta-analysis.

## 2. Methods

PubMed and Crossref were searched from database inception through 3 October 2026. The three conceptual searches were (1) (cattle OR cow OR dairy) AND (estrus OR oestrus) AND (acoustic OR sound OR vocalization OR vocalisation); (2) (sow OR pig OR porcine) AND (estrus OR oestrus) AND (acoustic OR sound OR vocalization OR vocalisation); and (3) (livestock OR cattle OR pig) AND (bioacoustic* OR vocalization OR vocalisation) AND (machine learning OR deep learning OR monitoring). In PubMed, these title/abstract searches returned 24, 8, and 41 records, respectively (73 records; 66 unique after removal of seven within-database overlaps). Parallel bibliographic searches in Crossref returned the first 100 relevance-ranked records per query (300 records; 283 unique). Ten records overlapped between databases, leaving 339 unique database records for screening.

Records were eligible for the core evidence set when they examined dairy cattle or sows and directly evaluated oestrus-related vocal behaviour, acoustic or multimodal oestrus recognition, or an acquisition, attribution, or validation step necessary for an oestrus monitoring pipeline. Engineering studies without an oestrus endpoint were retained only as supplementary evidence when they directly informed livestock sound acquisition, source localisation, denoising, deployment, or model validation. Records were excluded when they concerned unrelated species or reproductive states, lacked acoustic relevance, addressed non-reproductive vocal tasks without transferable methodological value, duplicated another report, or provided insufficient bibliographic or outcome information. English-language peer-reviewed journal articles were prioritised. Proceedings and book chapter sources [15,31,32,33,34] were used only as supporting technical evidence and were not treated as primary evidence for biological associations or reproductive outcomes.

After title and abstract screening of 339 unique database records, 24 reports were assessed in full and 16 directly relevant core records were retained. Backward citation searching and targeted retrieval of the biological, engineering, validation, and implementation literature added 78 supplementary sources, producing a final synthesis of 94 sources. Screening and extraction were conducted for a structured narrative review and were not performed independently in duplicate; this is now stated as a review-level limitation. During revision, the core study eligibility, reported metrics, labels, validation design, and publication type were rechecked against the cited reports.

Because species, housing, labels, sensors, sampling units, and validation designs were heterogeneous, performance metrics were not statistically pooled. Findings were organised through a common evidence chain comprising endocrine and behavioural plausibility, signal acquisition, event detection, reference standard definition, model validation, and translation to reproductive management. For comparable direct recognition studies, observed metric ranges are reported descriptively; they are not treated as pooled estimates. Software and device names reported below describe the cited studies; no new acoustic recordings or device experiments were conducted for this review.

## 3. Bioacoustic Foundations of Oestrus-Related Vocal Changes and Species Differences

### 3.1. Oestrus Behaviour, Endocrine Status, and Vocal Modifications

**Figure 1:**
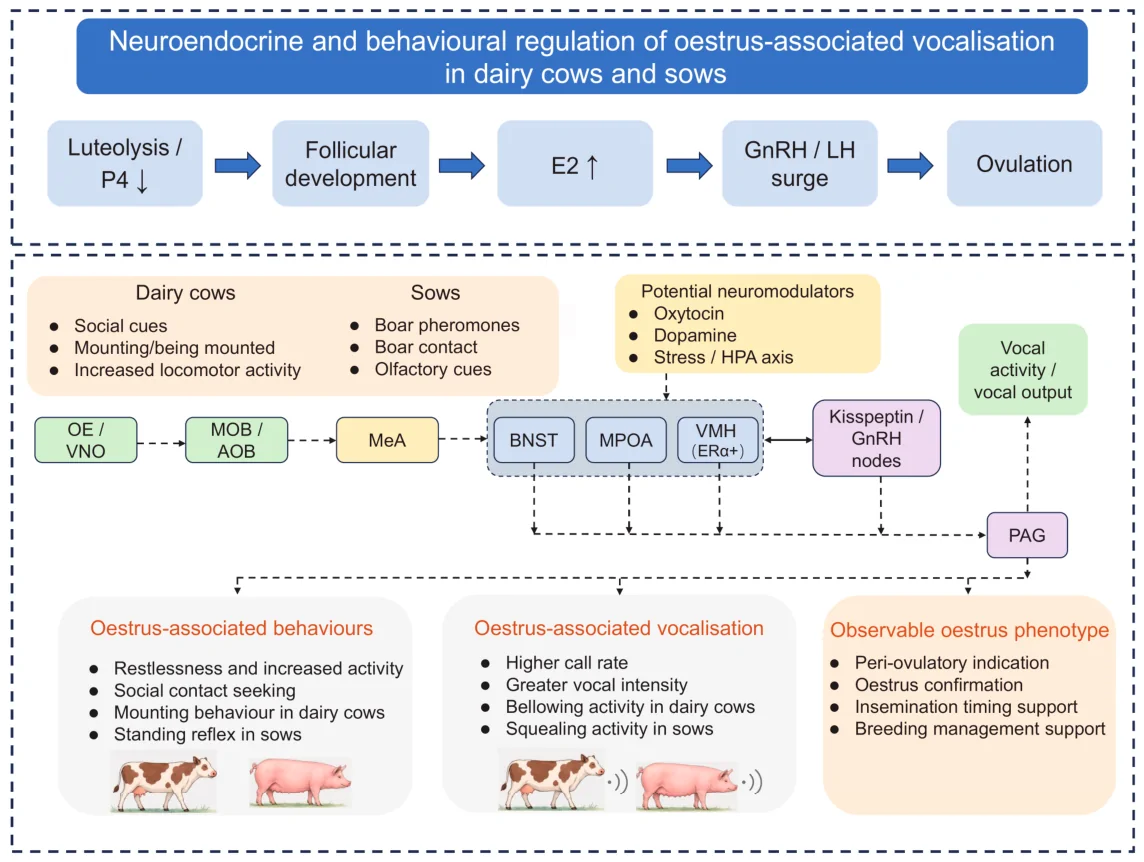
Conceptual pathways linking oestrus status, behavioural expression, and vocal changes in dairy cows and sows. Luteolysis, declining progesterone, follicular development, and rising oestradiol contribute to the gonadotrophin-releasing hormone and luteinising hormone surge and ovulation. These endocrine changes can promote oestrus-associated behaviours, including increased locomotor activity, mounting, or being mounted in dairy cows and boar-directed responses or standing reflexes in sows. Vocal changes may accompany these behavioural and motivational states, but the links between endocrine state, neural regulation, and specific acoustic variables remain indirect. Arrows indicate biologically plausible pathways rather than deterministic mechanisms. Blue boxes and solid blue arrows show the schematic endocrine sequence. Green denotes olfactory inputs and vocal output; yellow denotes the medial amygdala and potential neuromodulatory influences; pale blue and purple group central neural structures. Peach and grey backgrounds group species-specific cues and observable outcomes. Dashed black arrows denote hypothesised indirect links, and the double-headed arrow denotes a proposed reciprocal association. Colours identify functional groups rather than measured effect sizes. Abbreviations: AOB = accessory olfactory bulb; BNST = bed nucleus of the stria terminalis; E2 = oestradiol; ERα = oestrogen receptor alpha; GnRH = gonadotrophin-releasing hormone; HPA axis = hypothalamic–pituitary–adrenal axis; LH = luteinising hormone; MeA = medial amygdala; MOB = main olfactory bulb; MPOA = medial preoptic area; OE = olfactory epithelium; P4 = progesterone; PAG = periaqueductal grey; VMH = ventromedial hypothalamus; VNO = vomeronasal organ.

Vocal changes during oestrus are generally attributed to endocrine fluctuations, altered behavioural motivation, and social interactions. However, vocal signals do not map one-to-one onto specific internal states [25,26,27]. Source–filter theory distinguishes the fundamental frequency (*F*_0_), which reflects vocal fold vibration, from formants generated by vocal tract filtering. Both are also affected by body size, posture, mouth opening, and arousal [35]. Endocrine changes during the oestrous cycle influence behaviour, but causal links among hormones, neural pathways, and specific acoustic variables remain insufficiently established [36,37]. Figure 1 is therefore used as an organising model. The pathway from oestrus status to behavioural expression is biologically plausible, whereas the mapping from that state to any single acoustic variable remains indirect.

Mechanistically, luteolysis lowers progesterone restraint while follicular oestradiol rises and acts through hypothalamic and limbic circuits involved in sexual motivation, attention to social cues, locomotion, and receptivity [36,37]. Olfactory and vomeronasal inputs can further recruit the medial amygdala, bed nucleus of the stria terminalis, medial preoptic area, and ventromedial hypothalamus, with descending integration through the periaqueductal grey and brainstem vocal motor pathways. This sequence provides a plausible route from endocrine state to altered arousal, social approach, posture, respiratory drive, and calling probability. It does not, however, establish a unique hormonal signature in *F*_0_, formants, amplitude, or call duration; these acoustic variables are also affected by identity, body conformation, emotion, posture, distance, and the recording environment [25,26,27,35].

In dairy cows, the strongest direct evidence concerns vocalisation rate rather than a single diagnostic frequency threshold. Schön et al. [38] reported that total calls increased from peri-oestrous reference values of 28.0 ± 14.6 to 70.2 ± 19.4 calls/d before oestrus to 171.2 ± 61.5 calls/d at oestrus, before decreasing to 35.3 ± 11.4 calls/d after oestrus. Bellow and moo calls showed the same pattern, reaching 94.1 ± 39.7 and 77.1 ± 25.2 calls/d at oestrus, respectively. Röttgen et al. [39] found a similar temporal alignment between oestrous behaviour scores and vocalisation rates, with the highest natural oestrus vocalisation rate reaching 42.58 ± 21.89 calls/3 h before the oestrus climax, compared with 11.58 ± 5.51 calls/3 h under superovulation-related conditions. Individual attribution is also technically feasible under defined conditions: Röttgen et al. [40] reported caller identification in group-housed heifers with 87% sensitivity and 94% specificity. These findings support vocalisation rate and caller attribution as monitoring cues but not as standalone predictors of ovulation timing.

In sows, oestrous behaviour is closely linked to boar stimulation, the back-pressure response, and the standing reflex, while optimal insemination timing also depends on ovulation and reproductive tract physiology [5,41]. Available acoustic evidence is more limited than in dairy cows and is mainly based on call structure or stimulus-evoked responses. Jeon et al. [42] reported that oestrus-related sow vocalisations were louder and longer than general vocalisations, with intensity increasing from 69.2 ± 2.9 to 72.6 ± 1.9 dB, duration increasing from 1.0 ± 1.0 to 1.4 ± 0.4 s, and the fourth formant decreasing from 3.766 ± 0.271 to 3.598 ± 0.176 kHz. However, vocalisations were not significantly correlated with standing still during a back-pressure test (*r* = −0.22, *p* > 0.10), showing that sow acoustic cues should not replace the standing reflex or reproductive validation [43].

### 3.2. Acoustic Differences Between Dairy Cows and Sows

Evidence for vocalisation rate changes is more developed in dairy cows than in sows, although it remains based on a limited number of studies. In dairy cows, the main quantitative signal is a peri-oestrous rise in call rate, whereas in sows the clearer evidence concerns changes in call intensity, duration, and formant structure. Current reviews and modelling studies support the inclusion of acoustic or multimodal information in sow oestrus detection, but they do not establish fixed thresholds or precise insemination windows.

Dairy cows and sows differ in body size, vocal tract morphology, call repertoire, oestrous behaviour, and housing environment. Their acoustic features cannot therefore be evaluated using identical thresholds or model assumptions. Source–filter theory and emotional vocalisation research show that *F*_0_, formants, and spectral structure are jointly shaped by the vocal source, the vocal tract, and arousal state [35]. Oestrus-related changes in dairy cows are reported mainly as increased vocalisation rates and altered proportions of moo and bellow calls, whereas sow studies more often examine intensity, duration, formants, and call type composition. Table 1 therefore provides evidence for a shared concept of oestrus-related vocal modification but not for universal diagnostic thresholds. Acoustic findings in buffalo and other cattle breeds provide useful context but cannot be directly extrapolated to dairy cows or sows [6,31,44].

Despite morphological and environmental divergences, dairy cows and sows share a comparative biological foundation. Both species are mammals whose oestrus states encompass endocrine fluctuations, heightened behavioural motivation, altered social interactions, and specific management responses. These physiological and behavioural shifts may be indirectly reflected through vocalisation rates, call type ratios, call duration, or spectral structures. Thus, the similarity between the two species lies in the evidence chain demonstrating that oestrus states modify behaviour and vocal activity rather than in identical acoustic thresholds or specific frequency bands. For this reason, this review emphasises the comparability of vocal phenomena, detection pipelines, and validation logic rather than asserting uniformity in specific acoustic parameters.

From the perspective of acoustic mechanics, fundamental frequency, formants, sound pressure level, energy distribution, and spectral descriptors are shaped by vocal production, vocal tract filtering, posture, source distance, and background noise. In oestrus detection research, single acoustic parameters are best treated as candidate behavioural cues rather than deterministic markers of oestrus-related hormonal fluctuations [46].

Frequency ranges, sound pressure levels, call type ratios, call durations, and temporal variation are useful candidate inputs for acoustic oestrus recognition models. In dairy cows, sequence-level changes such as vocalisation rate and call type composition are especially relevant, whereas sow studies more often require integration with standing reflex testing, boar stimulation, thermal imaging, or video to interpret acoustic events within reproductive context.

### 3.3. Temporal Acoustic Features for Oestrus Stage Assessment

**Figure 2:**
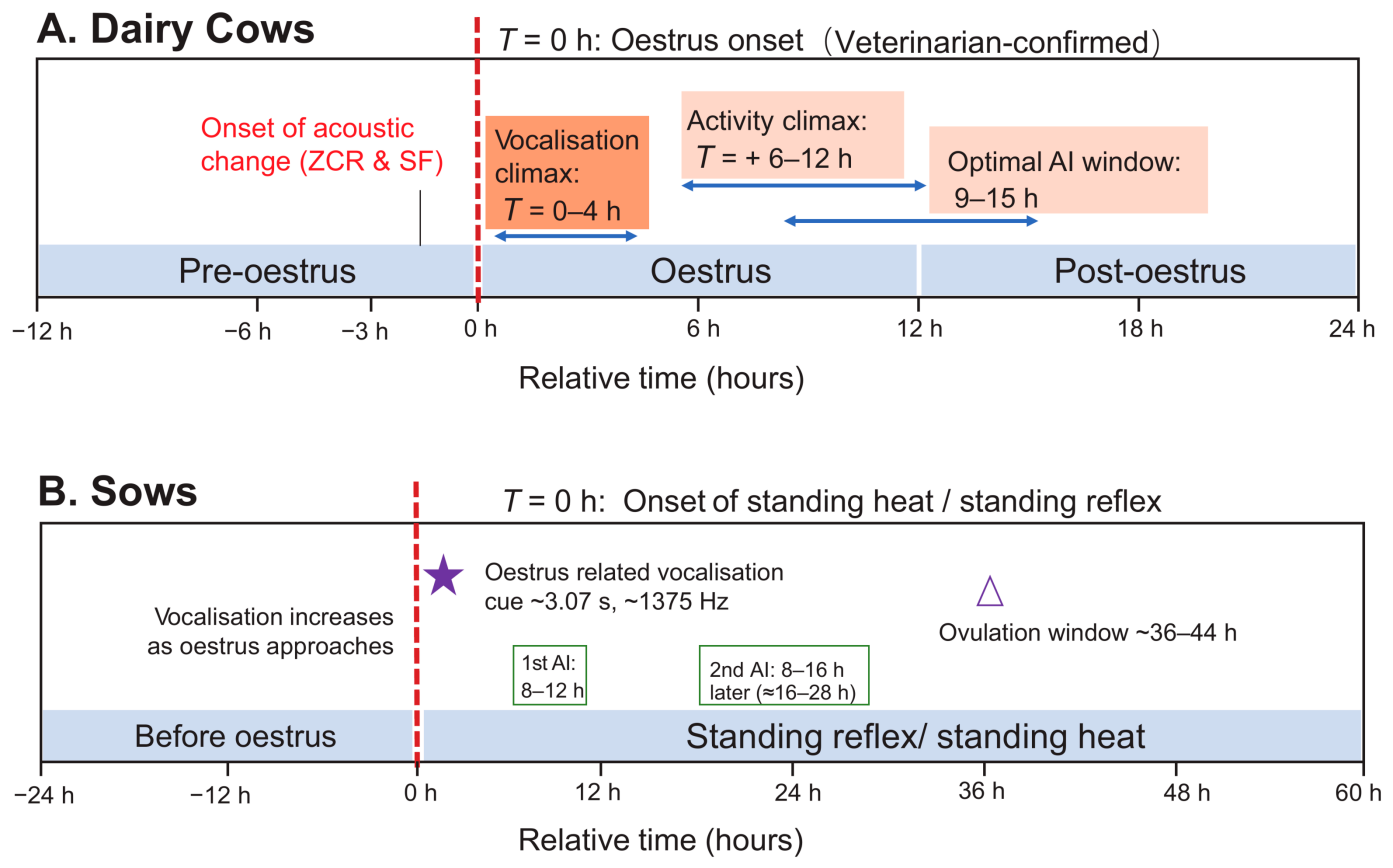
Schematic timing of acoustic cues, behavioural markers, insemination management and ovulation in dairy cows and sows. In cows, T = 0 h is oestrus onset, not oestrus climax. Röttgen et al. [39] observed the highest natural-oestrus vocalisation rate in the 3 h interval preceding the interval with the highest behavioural oestrus score. This climax-referenced result cannot be mapped to onset without a measured offset. In sows, T = 0 h is the first observed standing reflex or standing heat. Timing ranges and phase boundaries are schematic, not universal thresholds or acoustic predictions of ovulation or optimal insemination timing. Blue bands show behavioural phases; peach boxes and blue double-headed arrows show timing ranges. Red dashed lines mark onset; the purple star denotes a descriptive acoustic cue and the purple triangle a contextual ovulation window. Green boxes indicate insemination management timings. Abbreviations: AI = artificial insemination; SF = spectral flatness; ZCR = zero-crossing rate.

Figure 2 aligns acoustic observations with behavioural and management landmarks in dairy cows and sows. It is intended to compare temporal relationships rather than to present acoustically predicted insemination or ovulation windows, and the shown ranges need to be treated as indicative.

Oestrus is a temporally evolving behavioural and physiological state rather than an instantaneous event. Vocalisation rate per unit time, consecutive call counts, call type ratios, and temporal trends can therefore be more informative than the energy or frequency of an isolated call. For dairy cows, Figure 2 uses an onset-referenced schematic; the climax-referenced observations discussed above should not be interpreted as fixed delays from oestrus onset or as a universal acoustic threshold. For sows, Figure 2 anchors acoustic observations to the first observed standing reflex and displays later management and ovulation ranges as contextual landmarks. These landmarks do not establish that a call predicts ovulation or conception.

Acoustic oestrus detection can, consequently, progress beyond a simplistic binary oestrus/non-oestrus classification, but systems cannot claim precise ovulation or insemination time prediction without direct validation. A defensible workflow uses acoustic features to flag suspected oestrus states or stage transitions and then verifies them using activity, video-derived behaviour, back-pressure tests, reproductive records, or physiological measurements. Dairy cow monitoring studies show that activity, positioning, and reproductive records provide complementary information for oestrus identification [23,47]. Multimodal studies in sows and dairy cows likewise indicate that acoustic data should be fused with visual, thermal infrared, or other sensor data rather than carrying the full burden of an insemination decision [48].

## 4. Acquisition, Barn Acoustics, and Preprocessing of Acoustic Signals

The viability of acoustic oestrus detection depends on stable acquisition of target sounds under commercial conditions. Unlike controlled laboratory recordings, barn recordings are affected by variable call intensity, microphone placement, structural acoustics, reverberation, mechanical noise, and overlapping vocalisations [49]. An acquisition system is required to answer three operational questions: whether the target signal has an adequate signal-to-noise ratio, whether events can be attributed to an individual or pen, and whether the hardware can operate reliably under humidity, dust, cleaning, and animal interference [8,50].

Deployment strategy is shaped by species behaviour and housing. In dairy environments, acoustic tags, neck-mounted sensors, activity monitors, and positioning systems can form a distributed, individualised monitoring network. In sow environments, rooting, biting, impacts, cleaning, and pen-mate interactions can compromise wearable stability, making fixed, pen-level, array-based, or visual/radio-frequency identification (RFID)-integrated systems more practical. Commercial reproductive monitoring systems mainly rely on activity, rumination, RFID, boar contact, or electrical measurements, whereas acoustic oestrus detection remains concentrated in research systems such as the Cattle Call Monitor and dual-channel acoustic tags [40]. Thus, analytical pipelines can share common components, but hardware deployment, source attribution, and validation need species-specific designs.

### 4.1. Individualised Near-Field Acquisition and Wearable Acoustic Devices

**Figure 3:**
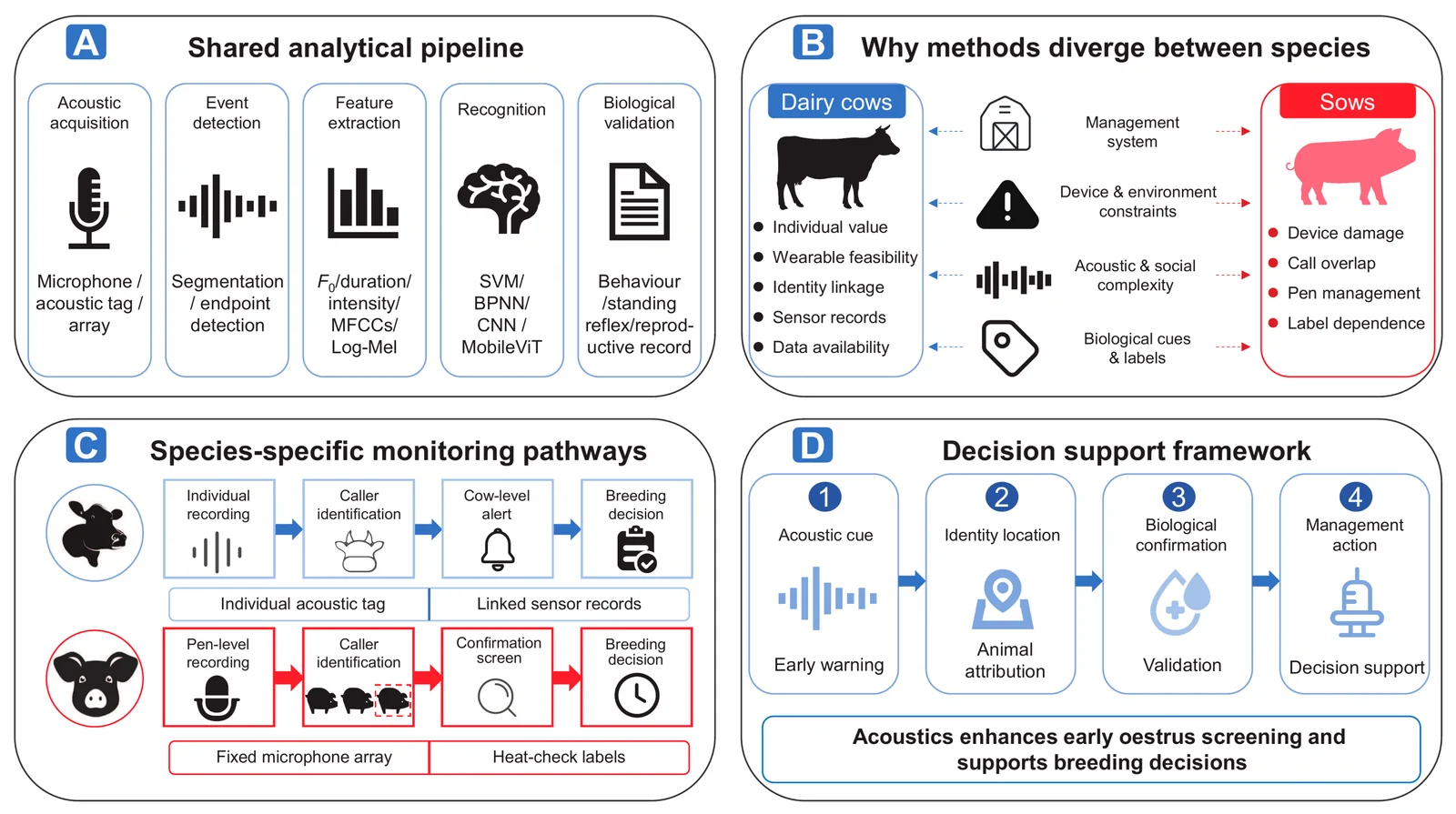
Conceptual framework for acoustic oestrus detection in dairy cows and sows. (**A**) Shared analytical pipeline from acoustic acquisition to event detection, feature extraction, recognition, and biological validation. (**B**) Main factors driving species-specific methodological differences, including individual management and sensor data availability in dairy cows and pen-level management, call overlap, and label dependence in sow systems. (**C**) Species-specific monitoring pathways. Dairy cow systems are more suited to individual acoustic tags, caller identification, and cow-level alerts, whereas sow systems more often require fixed microphone arrays, source attribution, and heat-check confirmation before service timing. (**D**) Decision support framework showing how acoustic cues, identity or location information, biological confirmation, and management actions can be integrated for reproductive decision support. Blue identifies dairy cow pathways and shared analytical or decision support components; red identifies sow-specific pathways. Solid arrows indicate processing or decision flow, and dashed arrows associate contextual constraints with each species. Colours distinguish schematic categories and do not indicate performance or evidence strength. Abbreviations: BPNN = back-propagation neural network; CNN = convolutional neural network; *F*_0_ = fundamental frequency; MFCCs = Mel-frequency cepstral coefficients; SVM = support vector machine.

Figure 3 makes the shared technical pipeline and the species-specific branches explicit. Both scenarios require acquisition, ground-truth construction, feature representation, preprocessing, recognition, and validation, but dairy cow systems more readily link signals to individuals, whereas sow systems more often depend on pen-level recording and behavioural confirmation.

The primary advantage of individualised near-field acquisition is the minimisation of the distance between the sound source and the microphone, which reduces the effects of far-field attenuation, reverberation, and background noise on target vocalisations [49]. For dairy cows, neck-mounted acoustic tags or dual-channel acoustic devices can be synchronised with activity, rumination, or spatial positioning data to support individual-level vocal recording and oestrus identification [18,51]. A dual-channel acoustic tag reached 91.25% accuracy and 98.83% precision for oestrous cow identification, and a selected-feature BPNN model reached 97.62% accuracy under the tested conditions [52]. These values show technical feasibility under defined conditions but should not be extrapolated to all commercial farms without cross-farm, cross-device, and long-term validation.

Individualised devices face stringent engineering constraints. Wearables need to balance weight, battery life, impact resistance, waterproofing, dustproofing, and animal comfort, a common bottleneck in long-term precision livestock sensing [28,29]. Proximity to the body does not confer absolute noise immunity. Friction, impacts, head shaking, and device movement can generate acoustic artefacts. This vulnerability is particularly acute in sow housing, where wearables are repeatedly exposed to rooting, biting, and group interactions. Commercial piggery conditions therefore require explicit tests of hardware durability and audio fidelity. Individualised near-field recording is thus most useful when the gain in target signal-to-noise ratio and individual attribution is supported by physical protection, artefact rejection, and validated long-term stability.

### 4.2. Fixed Microphones, Array Acquisition, and Individual Attribution

The installation height, array geometry, and localisation efficacy of fixed microphones are heavily dictated by barn architecture. Variations in ceiling height, wall materials, ventilation infrastructure, pen density, and cleaning methods require tailored engineering solutions, ranging from single suspended or wall-mounted microphones to linear and rectangular arrays. Traditional sound source localisation research confirms that array topology and spatial layout directly affect directional estimation and localisation stability [53,54,55]. While recent advances in machine learning and distributed arrays have enhanced localisation capabilities in complex scenes, these methods still require rigorous validation against severe barn reverberation and multi-source interference [56,57,58].

Fixed microphones and arrays eliminate the need for body attachment, thereby reducing the risks of device detachment, animal discomfort, and daily maintenance burdens. This non-contact approach is well-suited for sow pens, farrowing houses, gestation stalls, and large dairy free-stall barns [59]. Compared to wearable devices, fixed systems excel at capturing the long-term longitudinal data of the herd’s acoustic landscape. However, the major challenges for these systems remain individual attribution, reverberation management, and the resolution of multi-source audio aliasing [50]. While localisation and array methods provide directional or spatial cues, accurately resolving instances involving animal movement, physical occlusions, and simultaneous vocalisations typically mandates cross-validation with video surveillance, RFID, or manual verification [53,54,55].

For oestrus detection, a fixed system needs to do more than detect a sound; it should identify the relevant pen, attribute the event to an individual where required, and verify its association with oestrus. Without individual identity or pen-level localisation, even an accurate acoustic classifier cannot readily support breeding interventions. Dairy cow positioning and visual behaviour studies show that identity and behavioural semantics often require multi-source data fusion [47,60]. Fixed acoustic systems can, for this reason, integrate pen mapping, video tracking, RFID, or established manual verification workflows. Without precise localisation, their most defensible role is providing group-level or pen-level early warning followed by management confirmation.

The individual attribution challenge is particularly acute in sow environments. Group-housed sows or individuals in adjacent pens frequently vocalise simultaneously, and these calls can overlap with sounds generated by fighting, feeding, regrouping, or stress. A pen-level acoustic alert has limited reproductive value unless the detected event can be linked to the relevant animal, location, and oestrous stage. In sow facilities, pen-level alerts therefore require confirmation from localisation, RFID, video tracking, or routine heat-checking before they can guide individual breeding decisions. Dairy cow systems using individualised wearable devices can associate vocal events more directly with animal identity; their main barriers are artefact rejection, background noise control, and validation across farms.

### 4.3. Complex Barn Acoustics and Noise Typologies

Barn acoustic fields contain three broad classes of interference. Continuous mechanical noise from ventilation, manure scrapers, pumps, and feeding systems can mask target vocalisations in overlapping frequency bands [61,62]. Transient environmental noise, including metal impacts, high-pressure washing, and gate friction, can trigger broadband false detections [63,64]. Biological noise from non-target vocalisations, feeding, aggression, piglets, and herd movement is especially difficult to distinguish from target calls because it can resemble target call structure [7,65]. Table 2 shows that these are not minor background issues. Dairy free-stall barn recordings ranged from 52.5 to 83.0 dB, milking parlour noise ranged from 69 to 76 dB, and sow housing feeding or mating operations reached 104–115 and 94–115 dB, respectively [32,66].

The core challenge in acoustic oestrus detection is not simply the ambient noise level. It is the temporal, spectral, and semantic overlap between the target and interfering sources. In dairy barns, low-frequency oestrus bellows can overlap with fans, scrapers, and milking equipment. In sow facilities, high-frequency squeals can be mistaken for metal friction, piglet vocalisations, or stress-induced calls [69]. In one semi-open dairy barn analysis, representative median frequencies were 668.4 Hz for a cattle vocalisation and 780.9 Hz for an environmental sound, indicating that some target and background sounds can occupy adjacent frequency regions [68]. These failure modes cannot be resolved by a single high-pass filter or localisation algorithm. Reliability requires coordinated frontend hardware, preprocessing, event detection, semantic classification, and multimodal validation.

### 4.4. Sampling, Framing, Denoising, and Endpoint Detection

Within the preprocessing pipeline, sampling rate, framing, denoising, endpoint detection, and signal segmentation collectively determine subsequent feature quality. Acoustic preprocessing needs to balance computational load, bioacoustic feature fidelity, and cross-scenario robustness. For barn recordings, these steps not only affect model accuracy scores but also alter the stability of critical bioacoustic parameters, such as *F*_0_, formants, and call duration. Reported feature improvements or model performance metrics therefore need to be interpreted within the context of the task, acquisition environment, labelling standard, and data-partitioning method.

Sampling rate selection is constrained by target frequency bands and available deployment resources. Identifying low-frequency dairy cow bellows or calculating general vocalisation rates does not necessitate extremely high sampling rates. Conversely, analysing sow squeals, high-frequency harmonic structures, or individual voiceprints requires higher rates to preserve spectral details. However, increasing sampling rates concurrently increases storage requirements, transmission bandwidth, and edge computing load, refuting the assumption that higher is universally better.

Raw audio data generally require filtering, denoising, framing, endpoint detection, event segmentation, and feature standardisation prior to model ingestion. Time–frequency structures are typically represented using Short-Time Fourier Transforms (STFTs), Mel spectrograms, or Continuous Wavelet Transforms (CWTs). Short-time frame lengths and overlap rates need to be dynamically calibrated to balance target call duration with the required frequency resolution [70]. Endpoint detection and sound source separation are critical in group-housed environments where continuous audio streams may contain mechanical noise and overlapping biological sounds [69].

Crucially, denoising and signal reconstruction techniques, including spectral subtraction, Wiener filtering, adaptive filtering, Non-Negative Matrix Factorisation (NMF), and blind source separation, are required to support downstream state identification rather than merely enhancing human perceptual clarity [70,71,72]. Aggressive denoising frequently degrades the fundamental frequency, formants, harmonics, and nonlinear components inherent to the target call. Thus, evaluations of denoising techniques need to assess improvements in SNR alongside bioacoustic feature fidelity. Improvements in classification accuracy after denoising should be interpreted together with tests of feature preservation. Preprocessing that increases numerical performance but distorts biologically relevant variables, such as fundamental frequency, formants, call duration, or temporal call structure, may reduce the biological interpretability of the model.

Endpoint detection serves as the critical bridge between continuous audio streams and event-level recognition. Traditional methods based on short-time energy, zero-crossing rates, and spectral thresholds offer high computational efficiency for edge device screening, yet they are highly susceptible to false triggers in noisy barns. Deep learning-based sound classification models can extract complex acoustic patterns from spectrograms, but their reliability remains strongly dependent on annotation quality and cross-scenario testing [73]. While multi-stage or ensemble recognition models can mitigate specific noise artefacts, researchers need to verify explicitly whether these architectures fully preserve the precise onset, offset, duration, and acoustic structure of the target oestrus vocalisations [74].

## 5. Data, Features, and Models for Acoustic Oestrus Recognition

### 5.1. Data Acquisition and Oestrus Annotation Benchmarks

The performance of an acoustic oestrus model is constrained by the quality of its labels. Labels based only on stockperson observation are affected by observation schedules, observer experience, and missed nocturnal behaviour. Classical studies on dairy cows also reveal substantial variation in the duration and expression of oestrous behaviour [9,10]. Labels derived from synchronised video, activity sensors, back-pressure tests, ultrasonography, progesterone assays, or reproductive records may improve credibility, although they increase cost and operational complexity [11,12]. Some studies need to report whether boar stimulation, back-pressure testing, standing reflex confirmation, and explicit stage definitions were used [75].

Crucially, different oestrus annotation benchmarks are not interchangeable. Manual observation, video-derived behaviours, hormonal indicators, and ovulation confirmation need to be differentiated carefully during model evaluation. Manual observation aligns with routine farm management realities, whereas hormonal and ultrasound analyses provide physiological validation and back-pressure tests and standing reflexes reflect practical swine husbandry protocols [41]. Evidence that acoustic signals distinguish broad oestrus from non-oestrus periods should not be interpreted as evidence for ovulation prediction or optimal insemination timing. Claims about insemination windows require validation against stage-specific behavioural labels, endocrine profiles, ultrasound-confirmed ovulation, insemination records, or reproductive outcomes.

### 5.2. Class Imbalance and Data Augmentation

Acoustic oestrus datasets are typically highly imbalanced because non-oestrus recordings greatly outnumber true oestrus events and critical-stage samples. Event definitions, time-window length, and the composition of positive and negative samples can strongly affect reported performance [76]. A model may achieve high overall accuracy by predicting the majority non-oestrus class while failing to detect genuine events. Studies should therefore report class ratios, sample unit definitions, time-window parameters, and safeguards against leakage of recordings from the same animal across data splits.

Data augmentation techniques, including time stretching, pitch shifting, random cropping, background noise overlay, mixup, and time–frequency masking, are frequently used to mitigate sample scarcity. While animal audio classification and deep learning research heavily employ data augmentation to enhance model robustness, these techniques need to preserve bioacoustic plausibility [70,77]. Applying excessive pitch shifting or extreme time compression to oestrus calls risks obliterating species-specific identifiers or key oestrus-related acoustic features. Generative augmentation requires equally cautious application, demanding proof that synthetic samples do not introduce artefactual features [78,79]. While data augmentation remains a valuable research trajectory, asserting fixed-percentage performance improvements without task-specific empirical validation is methodologically unsound. Other livestock acoustic studies often utilised entropy, spectrograms, or deep learning models for welfare and state recognition. While providing technical frameworks, these methods do not constitute direct evidence for oestrus detection [80,81,82].

### 5.3. Acoustic Feature Representation

Table 3 groups candidate acoustic features into time-domain, frequency-domain, cepstral, and statistical families. These features describe different parts of an acoustic event and should not be interpreted as direct hormonal readouts. Fundamental frequency (*F*_0_), formants, and spectral descriptors are influenced by vocal fold vibration, vocal tract filtering, animal posture, emotional arousal, recording distance, and background noise. In this review, they are therefore treated as candidate behavioural cues rather than deterministic markers of oestrus-related endocrine status.

Acoustic features can be divided into event-level and sequence-level variables. Event-level features describe isolated calls through duration, energy, *F*_0_, formants, spectral centroid, bandwidth, zero-crossing rate, harmonic-to-noise ratio, and Mel-frequency cepstral coefficients (MFCCs). Sequence-level features describe vocalisation rates over defined windows, consecutive call counts, call type ratios, inter-call intervals, and temporal trends. For oestrus detection, sequence-level variables are particularly important because they better capture the time-dependent nature of oestrus behaviour.

Feature interpretability also differs across representations. Source–filter variables such as *F*_0_ and formants have clearer biological interpretation than many cepstral or deep learning representations, whereas MFCCs and spectrogram embeddings are often more useful for classification. Handcrafted features therefore remain valuable in limited acoustic oestrus datasets because they support model interpretation, error analysis, and cross-study comparison [83].

### 5.4. Feature Selection and Model Construction

Feature selection aims not only to reduce dimensionality but also to remove redundancy, improve generalisation, and preserve interpretable acoustic information. Filter methods efficiently remove low-variance or weakly associated features. Wrapper methods evaluate combinations via cross-validation, whereas embedded methods perform modelling and feature selection within random forests, gradient-boosting trees, or regularised models. Importantly, feature importance indicates statistical utility for classification, not biological causality. High predictive weights for vocalisation rate or specific MFCCs do not indicate that these variables cause or directly trigger oestrus-related physiological changes.

Model construction follows four broad pathways. Threshold rules and heuristic alerts are computationally efficient and suitable for low-power screening, but they are sensitive to individual variation and environmental noise [84]. Shallow models, including support vector machines, random forests, AdaBoost, and back-propagation neural networks, are useful for small or medium datasets with handcrafted features [85]. Deep and sequential architectures, including CNNs, LSTMs, Transformers, MobileNets, MobileViTs, and Conformers, can model complex spectrograms and temporal patterns but require larger datasets, high-quality labels, and rigorous partitioning [73,74]. Self-supervised learning, transfer learning, and domain adaptation may address label scarcity and cross-scenario generalisation, but their relevance to the final oestrus task needs to be explicitly demonstrated [69].

### 5.5. Model Evaluation Metrics and Validation Design

Model evaluation is a central weakness in much of the acoustic oestrus literature. Because the task is highly imbalanced and errors have different management costs, overall accuracy alone is inadequate. The reported values in Table 4 are promising but do not measure the same biological or management endpoint. They range from sound event detection and caller identification to binary oestrus classification, stage classification, and multimodal recognition. The table should therefore be read as a map of tasks and endpoints, not as a ranking of model performance.

Validation design often determines credibility more strongly than the choice of algorithm. Randomly shuffling audio segments can place correlated recordings from the same animal, day, or pen in both training and test sets, thereby inflating performance. More rigorous designs separate data by animal, pen, batch, farm, or non-overlapping time period. Precision livestock studies consistently emphasise the importance of cross-individual and cross-scenario validation [14]. Without cross-individual or cross-farm testing, a study demonstrates performance only within its local dataset and cannot establish readiness for commercial deployment.

Across the seven direct recognition model entries in Table 4 that reported accuracy, values ranged from 91.25% to 98.92%; among the four entries reporting F1-score, values ranged from 97.35% to 98.17%. These are descriptive observed ranges, not pooled estimates. No included study provided prospective cross-farm validation against ovulation, conception, farrowing, or another downstream reproductive outcome. Comparisons should therefore be based on the endpoint, reference standard, and split unit, not on the highest headline metric alone.

## 6. Multimodal Fusion, Representative Studies, and Application Translation

### 6.1. Complementarity of Acoustics with Activity, Vision, and Physiological Parameters

The primary value of acoustic signals lies in providing an observational dimension for oestrus detection that is distinctly separate from activity, visual, and physiological parameters. Research on dairy cow activity monitoring indicates that activity peaks offer critical cues for oestrus identification. However, these manifestations are not entirely consistent across diverse individuals and varying management conditions [93]. Acoustic studies suggest that a subset of dairy cows exhibits fluctuations in vocalisation rates around the oestrus climax. For this reason, vocalisations function most appropriately as supplementary indicators rather than standalone replacements for activity data or clinical judgement.

In commercial barns, microphones alone frequently fail to distinguish oestrus-related vocalisations from stress calls and background noise. A more defensible technical pathway uses acoustics as an early trigger or auxiliary source and then confirms events using activity sensors, computer vision, positioning data, or manual verification. For dairy cows, acoustic tags can be fused with activity, rumination, positioning, and reproductive records [48]. For sows, audio may be combined with thermal infrared imaging, video, boar stimulation, and the standing reflex [79]. The ambient sound environment can alter animal behaviour and physiological state, further complicating the interpretation of recorded audio [94]. Table 5 summarises complementary oestrus-monitoring modalities, their limitations, and their roles relative to acoustic monitoring.

### 6.2. Evidence Boundaries of Representative Studies

Representative dairy cow studies span early audio monitoring, peri-oestrus vocalisation rate observations, individual caller attribution, and dual-channel acoustic tag recognition. Reported examples include a 96.86% ODR, 7.46% FPR, and 3.14% FNR for an SVDD-based oestrus sound detection model [76], and 91.25% accuracy with 98.83% precision for dual-channel tag-based oestrous cow identification [52]. Because these studies differ in task definition, sample structure, equipment, and validation design, their results demonstrate application potential rather than generalisable commercial performance.

Evidence from the authors’ research programme [48,52,85] is therefore interpreted alongside independent groups. Independent studies have reported peri-oestrous vocalisation changes and caller attribution in dairy cattle [38,39,40], SVDD-based oestrus sound detection [76], and sow acoustic, audio–thermal, and stage recognition models [86,87,88,89,90]. Agreement across groups supports technical plausibility, but the absence of prospective, independent cross-farm reproductive outcome studies remains the decisive limitation.

Sow studies also show active development of acoustic and multimodal recognition. Reported examples include 97.52% accuracy and 97.59% F1-score for oestrus vs. non-oestrus sound classification [86] and 98.92% accuracy with 97.35% F1-score for audio–thermal multimodal recognition [87]. These results remain dependent on label quality and endpoint definition. The strongest reproductive benchmarks are still the standing reflex, ovulation confirmation, and actual insemination outcomes.

## 7. Discussion

### 7.1. Translational Limits of Acoustic Oestrus Detection in Dairy Cows and Sows

Acoustic oestrus detection in dairy cows and sows should be interpreted as two species-specific applications within a shared evidence framework. In dairy cows, the strongest quantitative evidence concerns sequence-level vocal activity rather than a fixed diagnostic frequency. Total calls increased from 28.0 ± 14.6 calls/d during the reference period and 70.2 ± 19.4 calls/d before oestrus to 171.2 ± 61.5 calls/d at oestrus, before decreasing to 35.3 ± 11.4 calls/d after oestrus [38]. This pattern supports the biological relevance of vocalisation rate, call type ratio, and temporal clustering as candidate cues for oestrus detection.

In sows, the available evidence is less mature and points more strongly to call structure changes than to vocalisation rate thresholds. Oestrus-related vocalisations were louder and longer than general vocalisations, with intensity increasing from 69.2 ± 2.9 to 72.6 ± 1.9 dB and duration increasing from 1.0 ± 1.0 to 1.4 ± 0.4 s [42]. However, vocalisation was not significantly correlated with standing still during back-pressure testing (*r* = −0.22, *p* > 0.10), indicating that sow acoustic cues should be treated as auxiliary behavioural information rather than as replacements for the standing reflex or reproductive validation [43]. Therefore, the similarity between dairy cows and sows lies in the evidence chain that oestrus modifies vocal behaviour, not in shared diagnostic thresholds.

Representative model metrics support technical feasibility but not commercial readiness. As summarised in Table 4, reported studies include sound event detection, caller identification, binary oestrus classification, oestrus stage classification, and audio–thermal multimodal recognition, but these endpoints are not equivalent biological or management tasks. The main limitations are therefore not only model accuracy, but also the biological meaning of the label, the independence of the validation, and the proximity of the endpoint to a reproductive decision.

### 7.2. Commercial Barriers and Future Validation Priorities

Commercial barn acoustics remain a major barrier to reliable acoustic oestrus detection. The reviewed studies show that dairy and sow housing environments are affected by a range of sounds from moderate barn noise to high-intensity operational noise, with reported values extending from 52.5 dB in dairy free-stall recordings to more than 100 dB during some sow housing activities [32,66]. These levels can mask target vocalisations, generate false triggers, and reduce the usefulness of recordings collected during feeding, cleaning, mating, or milking-related operations. Future studies should therefore report not only classification accuracy, but also signal-to-noise ratio, microphone position, recording distance, barn operation status, and the extent to which denoising preserves *F*_0_, formants, call duration, and temporal call structure.

Hardware deployment and animal attribution determine whether acoustic outputs can support reproductive decisions. Dairy cow systems can more readily associate vocal events with individuals through collars, ear tags, positioning systems, or near-field acoustic tags. This makes dairy cow acoustic monitoring more suitable for individualised alerts, especially when acoustic data are fused with activity, rumination, positioning, and reproductive records. In contrast, sow production is commonly organised around pens, batches, and routine heat-checking. A pen-level acoustic alert has limited reproductive value unless the call can be linked to the relevant sow and confirmed against boar stimulation, back-pressure testing, or the standing reflex. For sow systems, the priority should therefore be fixed microphones, source localisation, video or radio-frequency identification confirmation, and validation against practical reproductive endpoints.

Inconsistent oestrus labels remain a central methodological limitation. Manual observation, video-derived behaviour, activity peaks, progesterone assays, ultrasonography, back-pressure tests, standing reflexes, and reproductive outcomes do not represent equivalent ground truths. Evidence that a model detects oestrus-associated sounds should not be interpreted as evidence that it predicts ovulation, optimal insemination timing, or conception. Future studies should state explicitly whether the target is a vocalisation type, a general oestrus state, a specific oestrus stage, the standing reflex, ovulation, or an insemination decision. Validation should separate data by animal, pen, batch, farm, or non-overlapping time period rather than randomly splitting correlated audio segments from the same animal or environment.

At present, commercial reproductive monitoring is more advanced for activity, rumination, positioning, visual observation, boar contact, and herd management records than for acoustic-only oestrus detection. This gap indicates that the near-term commercial route for acoustics is unlikely to be a standalone replacement for existing systems. A more realistic pathway is to use acoustic monitoring as an additional layer within established decision support platforms. Microphones or acoustic tags can provide early warning, while activity sensors, video, radio-frequency identification, heat-checking, and reproductive records confirm the animal identity and management action. For dairy cows, the commercial value should be tested by whether acoustic information reduces missed oestrus events, shortens verification time, or improves insemination timing across farms and devices. For sows, commercial progress depends on robust pen-level recording, source attribution, compatibility with routine heat-checking, and validation against the standing reflex, conception rate, non-productive days, and labour requirements.

Economic feasibility should be evaluated at the system level rather than inferred from model accuracy. Studies should report microphone or tag cost, installation, network and storage requirements, cleaning and replacement, calibration and maintenance, integration with herd management software, false-alert verification labour, and staff training. Benefits should be measured as time saved, missed oestrus events avoided, changes in days open or sow non-productive days, conception or farrowing outcomes, and cost per monitored animal or pen. Adoption will also depend on alert interpretability, compatibility with existing workflows, data ownership, and whether the system remains useful when connectivity or one sensor stream fails [2,3,4,20,30].

Recent work also illustrates the speed of model development. BAE-LiteNet combines diffusion-based augmentation with a lightweight behaviour-aware network and reported 98.18% accuracy and a 98.17% F1-score on 4400 labelled sow vocalisations [90]. These results require independent, animal-level, and cross-farm validation before they can support breeding decisions.

### 7.3. Review-Level Limitations

This review has four methodological constraints: First, the Crossref retrieval was limited to the first 100 relevance-ranked results per query and cannot guarantee exhaustive engineering coverage. Second, backward citation and targeted contextual sources formed an open supplementary stream rather than a closed systematic search denominator. Third, screening and extraction were not independently duplicated, and a formal risk-of-bias tool was not applied. Fourth, heterogeneous species, labels, sampling units, and validation designs precluded meta-analysis. The resulting framework should therefore be read as an analytically structured signpost to the evidence and its gaps, not as a definitive effect estimate or systematic ranking of technologies.

## 8. Conclusions and Research Agenda

### 8.1. Conclusions

Acoustic monitoring provides a plausible non-contact layer for reproductive management, but the strength and meaning of the evidence differ between dairy cows and sows. Dairy cow studies most consistently support peri-oestrous changes in vocalisation rate and technically feasible caller attribution; sow studies more often support call structure changes and multimodal recognition linked to boar stimulation or the standing reflex. High internal model metrics do not demonstrate prediction of ovulation, optimal insemination timing, or commercial reproductive benefit. The defensible near-term role of acoustics is therefore to provide early warning and behavioural verification within a multimodal decision process. Species-specific deployment, explicit reference standards, leakage-resistant validation, and economic evaluation are required before routine adoption.

### 8.2. Priority Research Agenda

1. Standardise labels and reference standards. Report whether the target is a call type, behavioural oestrus, standing heat, an oestrus stage, ovulation, or an insemination decision, and document who or what established the label.
2. Prevent information leakage. Partition evaluation data by animal and, where relevant, by pen, batch, recording session, and non-overlapping time period; report these units explicitly.
3. Test cross-farm and cross-device robustness. Conduct prospective external validation across housing types, breeds, microphones or tags, seasons, and operational noise conditions before commercial claims are made.
4. Build calibrated multimodal decision support. Fuse acoustics with activity, vision, identity, physiological, or management data; quantify uncertainty and route uncertain alerts to human confirmation rather than forcing a binary decision.
5. Measure adoption and economic value. Report total system cost, maintenance, false-alert labour, workflow fit, time saved, missed heats, days open or non-productive days, and conception or farrowing outcomes.
6. Establish open benchmarks and reporting standards. Release de-identified audio, labels, metadata, split definitions, and code where ethically and commercially feasible, and use shared reporting checklists to enable reproducible comparison.

## Figures and Tables

**Table 1:** Quantitative evidence for oestrus-related vocal changes in dairy cows and sows.

Species	Condition	Comparison	Endpoint	Quantitative Result	Statistics	Reference Standard/Ground Truth
Dairy cow	Peri-oestrous period	Before, during, and after oestrus	Total calls	Before: 28.0 ± 14.6 to 70.2 ± 19.4 calls/d; oestrus: 171.2 ± 61.5 calls/d; after: 35.3 ± 11.4 calls/d	—	Study-defined peri-oestrous periods based on behavioural and clinical reproductive assessment
Dairy cow	Peri-oestrous period	Before, during, and after oestrus	Bellow calls	Before: 9.1 ± 4.8 to 34.3 ± 12.2 calls/d; oestrus: 94.1 ± 39.7 calls/d; after: 13.9 ± 6.3 calls/d	—	Study-defined peri-oestrous periods based on behavioural and clinical reproductive assessment
Dairy cow	Peri-oestrous period	Before, during, and after oestrus	Moo calls	Before: 14.7 ± 7.1 to 35.9 ± 9.0 calls/d; oestrus: 77.1 ± 25.2 calls/d; after: 21.4 ± 5.4 calls/d	—	Study-defined peri-oestrous periods based on behavioural and clinical reproductive assessment
Dairy cow	Oestrus climax	Call type distribution	Moo calls	45.0 ± 6.5% of calls at oestrus	—	Behaviourally and clinically defined oestrus climax
Dairy cow	Oestrus climax	Call type distribution	Bellow calls	55.0 ± 6.5% of calls at oestrus	—	Behaviourally and clinically defined oestrus climax
Dairy cow	Post-oestrus period	Oestrus vs. after oestrus	Total calls	171.2 ± 61.5 to 35.3 ± 11.4 calls/d	—	Study-defined post-oestrous period
Dairy cow	Natural oestrus	3 h window before oestrus climax	Vocalisation rate	42.58 ± 21.89 calls/3 h	Significant	Maximum behavioural oestrus score during natural oestrus
Dairy cow	Natural vs. superovulated oestrus	Natural oestrus vs. superovulation-related condition	Vocalisation rate	42.58 ± 21.89 vs. 11.58 ± 5.51 calls/3 h	Significant	Maximum behavioural oestrus score; natural vs. superovulation-related condition
Dairy cow	Oestrus monitoring	Vocalisation rate and behavioural score	Temporal pattern	Vocalisation rate and oestrous behaviour score followed similar peri-oestrous trajectories	—	Behavioural oestrus score aligned with vocalisation observations
Dairy cow	Group-housed heifers	Individual recording under group conditions	Caller identification	Sensitivity 87%; specificity 94%; PPV 96%; NPV 80%	—	Video-confirmed caller identity; not an oestrus classifier
Sow	Oestrus-related vocalisations	General vs. oestrus-related calls	Intensity	69.2 ± 2.9 vs. 72.6 ± 1.9 dB	*p* < 0.001	Study-defined general vs. oestrus-related calls; physiological confirmation not reported
Sow	Oestrus-related vocalisations	General vs. oestrus-related calls	Duration	1.0 ± 1.0 vs. 1.4 ± 0.4 s	*p* < 0.05	Study-defined general vs. oestrus-related calls; physiological confirmation not reported
Sow	Oestrus-related vocalisations	General vs. oestrus-related calls	Fourth formant	3.766 ± 0.271 vs. 3.598 ± 0.176 kHz	*p* < 0.01	Study-defined general vs. oestrus-related calls; physiological confirmation not reported
Sow	Oestrus-related vocalisations	General vs. oestrus-related calls	Vocalisation types	Five general vocalisation types and three oestrus-related vocalisation types	—	Study-defined general vs. oestrus-related calls; physiological confirmation not reported
Sow	Oestrus sow vocalisation	Descriptive acoustic characterisation	Call duration	Approximately 3.07 s	—	Descriptive vocalisation recorded under husbandry-defined oestrus
Sow	Oestrus sow vocalisation	Descriptive acoustic characterisation	Main energy resonance frequency	Approximately 1375 Hz	—	Descriptive vocalisation recorded under husbandry-defined oestrus
Sow	Back-pressure test	ISO control vs. odour-treated groups	Vocalisation occurrence	ISO 41.4%; BSA 76.1%; quinoline 84.4%; androstenone 66.1%; androstenol + quinoline 69.6%	—	Back-pressure test under control or odour treatment
Sow	Back-pressure test	Vocalisation vs. standing still	Association with standing reflex	*r* = −0.22	*p* > 0.10	Standing-still response during back-pressure testing

Abbreviations: BSA, boar salivary androstenone; dB, decibel; ISO, control odour; kHz, kilohertz; NPV, negative predictive value; PPV, positive predictive value. Sources: Schön et al. [38], Röttgen et al. [39], Röttgen et al. [40], Jeon et al. [42], Xin and DeShazer [45], and McGlone et al. [43]. A dash indicates that the statistical test or exact probability value was not reported in the cited source. Values are reported as presented in the cited studies and should not be interpreted as universal diagnostic thresholds. Ground-truth descriptions reflect the original reports and are not equivalent across studies.

**Table 2:** Barn noise and acoustic interference relevant to oestrus-related vocalisation detection.

Housing or System	Sound Source or Method	Value or Method	Relevance
Dairy cow housing	General barn noise	61–73 dB	Background noise
Free-stall dairy barn	Day-time background noise	52.5–83.0 dB	Variable recording background
Milking parlour	Annual mean noise	69–76 dB	Equipment and handling noise
Herringbone parlour	Milking parlour noise	Mean 69.2 dB; maximum 82 dB	Call masking
Dairy cow acoustic tag	Environmental noise channel	Omnidirectional microphone used as reference	Noise reference
Dairy cow acoustic tag	Noise reduction method	LMS adaptive filtering with wavelet thresholds	Noise reduction
Semi-open dairy barn	Cattle vocalisations	Median frequency 668.414 Hz	Target range
Semi-open dairy barn	Environmental sounds	Median frequency 780.885 Hz	Frequency overlap
Semi-open dairy barn	Bird sounds	Median frequency 3.83 kHz	Biological noise
Semi-open dairy barn	Machine sounds	Median frequency 4.424 kHz	Mechanical noise
Sow housing	Mechanical ventilation	Approximately 73 dB	Continuous background noise
Sow housing	Natural ventilation	Approximately 10 dB lower than mechanical ventilation	Ventilation-dependent background
Sow housing	Farm sound frequency range	20 Hz to 6.3 kHz	Wide-band overlap
Sow housing	Feeding	104–115 dB	High-level masking
Sow housing	Mating operation	94–115 dB	Management noise
Sow housing	High-pressure cleaning	Approximately 105 dB	Unsuitable recording period
Sow housing	Feed mixing	88–93 dB	Mechanical noise

Abbreviations: dB, decibel; Hz, hertz; kHz, kilohertz; LMS, least mean squares. Sources: Venglovsky et al. [32], Sistkova et al. [67], Özmen et al. [68], Wang et al. [52], and Dimov et al. [66]. Values are reported as presented in the cited studies. Sow housing noise data are used as reference values where oestrus-specific noise measurements were unavailable.

**Table 3:** Candidate acoustic features for acoustic oestrus detection in dairy cows and sows.

**Type**	**No.**	**Feature**	**Definition**	Equation
Time domain	1	Zero-crossing rate	Proportion of adjacent samples with opposite signs; indicates high-frequency or noisy content.	$ZCR=\frac{1}{N-1}\sum_{n=1}^{N-1}I\left(x_{n}x_{n-1}<0\right)$
	2	Root mean square amplitude	Square root of mean squared amplitude; an amplitude descriptor related to acoustic energy.	$RMS=\sqrt{\frac{1}{N}\sum_{n=0}^{N-1}{x_{n}}^{2}}$
	3	Duration	Time interval between onset and offset of a single extracted vocalisation.	$Duration=t_{end}-t_{start}$
	4	Mean signal amplitude	Arithmetic mean of signed sample amplitudes in the analysis window; describes the signal offset rather than acoustic loudness.	$\mu =\frac{1}{N}\sum_{n=0}^{N-1}x_{n}$
	5	Signal variance	Mean squared deviation of sample amplitudes from the frame mean; describes amplitude variability.	$\sigma^{2}=\frac{1}{N}\sum_{n=0}^{N-1}{\left(x_{n}-\mu \right)}^{2}$
	6	Kurtosis	Fourth central moment divided by the squared variance; measures sensitivity to extreme amplitude values (raw kurtosis).	$Kurtosis=\frac{\frac{1}{N}\sum_{n=0}^{N-1}{\left(x_{n}-\mu \right)}^{4}}{\sigma^{4}}$
	7	Autocorrelation function	Unnormalised sum of products of the signal and its lagged copy; used to assess periodicity.	$R\left(l\right)=\sum_{n=0}^{N-1-l}x_{n}x_{n+l}$
Spectral domain	8	Spectral centroid	Energy-weighted mean frequency of the spectrum; describes spectral brightness.	$SC=\frac{\sum_{k=0}^{K-1}f_{k}E_{k}}{\sum_{k=0}^{K-1}E_{k}}$
	9	Spectral roll-off frequency	Frequency of the first bin at which cumulative spectral power reaches 85% of the total.	$f_{roll}=f_{k_{85}}$
	10	Spectral bandwidth	Spread of spectral energy around the centroid; indicates frequency dispersion.	$BW=\sqrt{\frac{\sum_{k=0}^{K-1}{\left(f_{k}-SC\right)}^{2}E_{k}}{\sum_{k=0}^{K-1}E_{k}}}$
	11	Spectral flatness	Degree to which the spectrum is noise-like rather than tone-like.	${SF}_{dB}=10{log}_{10}\frac{{\left(\prod_{k=0}^{K-1}E_{k}\right)}^{\frac{1}{K}}}{\frac{1}{K}\sum_{k=0}^{K-1}E_{k}}$
	12	Fundamental frequency	Fundamental frequency estimated from the period of a voiced call; not defined for aperiodic calls.	$F_{0}=\frac{f_{s}}{\tau_{0}}$
Cepstral features	13	Mel-frequency cepstral coefficients	Compact representation of the Mel-scaled spectral envelope of a vocalisation.	$c_{q,t}=\sum_{m=0}^{M-1}U_{m,t}cos\left(\frac{\pi q\left(m+0.5\right)}{M}\right)$
	14	First-order MFCC difference	Regression estimate of MFCC change across neighbouring frames; captures short-term spectral dynamics.	$d_{q,t}=\frac{\sum_{h=1}^{H}h\left(c_{q,t+h}-c_{q,t-h}\right)}{2\sum_{h=1}^{H}h^{2}}$
	15	Second-order MFCC difference	Regression estimate of change in first-order MFCC differences across neighbouring frames.	$\Delta^{2}c_{q,t}=\frac{\sum_{h=1}^{H}h\left(d_{q,t+h}-d_{q,t-h}\right)}{2\sum_{h=1}^{H}h^{2}}$
Call-level statistics	16	Vocalisation frequency within 1 h	Number of detected vocalisations within a 1 h recording window.	$N_{1h}=\sum_{i}I\left(t_{i}\in [T_{0},T_{0}+1h)\right)$
	17	Proportion of low-pitched vocalisations within 3 h	Low-pitched vocalisations divided by all detected vocalisations within a 3 h window.	$PoM=\frac{N_{low,3h}}{N_{all,3h}}$
	18	Number of continuous vocalisations within 1 h	Number of calls belonging to sequences of at least two calls within a 1 h window.	$C_{1h}=\sum_{j}n_{j}I\left(n_{j}\geq 2\right)$
	19	Maximum number of continuous vocalisations	Largest number of consecutive calls within one vocalisation sequence.	$C_{max}=\underset{j}{max}n_{j}$

Abbreviations: ACF, autocorrelation function; BW, spectral bandwidth; *F*_0_, fundamental frequency; MFCCs, Mel-frequency cepstral coefficients; RMS, root mean square amplitude; SC, spectral centroid; SF, spectral flatness; ZCR, zero-crossing rate. Symbols: *x_n_*, sample amplitude; *N*, samples per frame; μ and σ^2^, frame mean and population variance; ℓ, non-negative sample lag less than *N*; *K*, spectral bins; *f_k_* and *E_k_*, bin frequency and non-negative power; *f*_roll_, 85% spectral roll-off frequency; *k*_85_, first bin index at which cumulative power reaches 85% of the total; *f*_s_, sampling rate; *τ*_0_, estimated fundamental period in samples; *c_q,t_*, MFCC *q* in frame t; *U_m,t_*, log energy from Mel filter m in frame t; *M*, Mel filters; *H*, delta-regression half-window; *d_q,t_*, first-order MFCC difference. For MFCCs, *q* = 0, …, *M* − 1; boundary frames are repeated when computing differences, with an integer regression half-window *H* ≥ 1. The fundamental-period estimate must satisfy *τ*_0_ > 0. Zero-crossing rate requires *N* ≥ 2; kurtosis requires positive variance. Spectral ratios require a non-zero denominator; a positive numerical floor is needed for zero-valued bins in log or product operations. *T*_0_ is the counting-window start and *t_i_* is call onset time; I is the indicator function. PoM is undefined when no calls occur. Within a 1 h window, *G_j_* is a maximal sequence of calls with an offset-to-next-onset gap of less than 1 s and *n_j_* is its call count; *C*_1h_ counts calls in sequences with *n_j_* ≥ 2, and *C*_max_ is zero when no calls occur. The 1 s gap is illustrative, not a universal biological threshold. Features are candidate acoustic cues or model inputs, not direct hormonal markers of oestrus.

**Table 4:** Published acoustic and multimodal recognition studies and technical enablers relevant to oestrus monitoring, with explicit ground-truth and validation interpretation.

Study/Task and Ground Truth	Validation Design and Reported Result	Interpretation, Publication Type, and Ref.
Dairy cow; Cattle Call Monitor caller identification. Ground truth: video-confirmed caller identity.	Pilot evaluation in group-housed heifers in the same research setting. Sensitivity 87%; specificity 94%; PPV 96%; NPV 80%.	Attribution task, not oestrus classification; no external farm test. Peer-reviewed journal [40].
Dairy cow; SVDD oestrus sound detection. Ground truth: visual standing heat plus subsequent pregnancy confirmation.	100 oestrus and 180 normal sounds; random 80% training repeated five times. ODR 96.86%; FPR 7.46%; FNR 3.14%.	Random sound-level splitting can share animal/environment information; no cross-farm test. Peer-reviewed journal [76].
Dairy cow; dual-channel acoustic tag individual recognition. Ground truth: study-specific oestrus labels.	Within-study evaluation; independent farm/device test not reported. Accuracy 91.25%; precision 98.83%; sensitivity 91.75%; specificity 83.68%.	Generalisation beyond sampled animals/settings is unknown. Peer-reviewed journal [52].
Dairy cow; BPNN with selected acoustic features. Ground truth: study-specific oestrus labels.	Within-study feature/model evaluation. Accuracy 97.62%; precision 98.07%; sensitivity 97.17%; specificity 97.19%; F1 97.63%.	Potential optimism without animal-, time-, and farm-independent testing. Peer-reviewed journal [52].
Dairy cow; soundscape separation. Ground truth: cattle vs. environmental sound categories.	Descriptive semi-open barn recordings; no reproductive classification metric.	Engineering context only; not an oestrus endpoint. Peer-reviewed journal [68].
Cattle/calf; Hilbert follower and HMM call event detection. Ground truth: annotated cattle or calf calls.	Fixed recording unit evaluation; automatic call detection demonstrated.	Transfer to oestrus calls/farms not established. Conference proceeding [33].
Sow; MobileNetV3_esnet binary recognition. Ground truth: oestrus vs. non-oestrus sound labels.	Internal dataset split; no external farm test. Precision 97.12%; recall 97.34%; F1 97.59%; accuracy 97.52%; model size 5.94 MB.	Correlated clips may inflate performance if animals/sessions overlap. Peer-reviewed journal [86].
Sow; APO-CViT audio–thermal recognition. Ground truth: study-defined multimodal oestrus labels.	Internal audio–thermal dataset evaluation. Accuracy 98.92%; recall 95.83%; F1 97.35%.	No independent cross-farm reproductive outcome validation. Peer-reviewed journal [87].
Sow; UM-ASPP-MobileViT four-stage classification. Ground truth: pro-oestrus, oestrus, post-oestrus, and non-oestrus labels.	Internal dataset evaluation; accuracy 96.52%.	Stage labels and split unit constrain interpretation. Peer-reviewed journal [88].
Sow; stacked LSTM stage/noise classification. Ground truth: study-defined stage and noise labels.	Internal/offline and field-oriented evaluation. Accuracy 96.17%; latency 41 ms.	No cross-farm reproductive outcome validation. Peer-reviewed journal [89].
Sow; BAE-LiteNet with diffusion augmentation. Ground truth: labelled sow vocalisation classes.	Internal evaluation on 4400 labelled vocalisations. Accuracy 98.18%; F1 98.17%.	Augmented/correlated samples require animal-level independent testing. Peer-reviewed journal [90].
Sow; deep learning audio diagnosis support. Ground truth: study-defined oestrus status.	Technical demonstration; no comparable reproductive metric reported.	Supporting evidence; validation independence unclear. Conference proceeding [34].
Sow housing; microphone array/sound camera. Ground truth: spatial source or visualised sound event.	Pen-level engineering evaluation; localisation/visualisation demonstrated.	Enabling technology only; not an oestrus endpoint. Peer-reviewed journals [91,92].

Abbreviations: APO-CViT, adaptive particle optimisation convolutional vision Transformer; BPNN, back-propagation neural network; FNR, false-negative rate; FPR, false-positive rate; HMM, hidden Markov model; MFCC, Mel-frequency cepstral coefficient; ODR, oestrus detection rate; PPV, positive predictive value; SVDD, support vector data description. Conference proceedings are retained as supporting technical evidence and are not given the same evidential weight as peer-reviewed biological or reproductive validation studies.

**Table 5:** Complementary oestrus monitoring modalities and their roles relative to acoustics.

Modality and Target	Strengths and Limitations	Role Relative to Acoustics
Visual observation/back-pressure testing: mounting, standing heat, and sow standing reflex	Direct behavioural interpretation and familiar workflow; limited by labour, observer variability, and brief/nocturnal events.	Confirms whether an acoustic alert corresponds to recognised oestrus behaviour.
Activity, rumination, or location sensing: locomotion, feeding, rumination, and spatial behaviour	Continuous individual monitoring with mature dairy deployment; weak/silent heats, attachment effects, and false alerts remain.	Provides an independent temporal signal for alert fusion [13,14,15,22,23].
Progesterone and reproductive physiology: endocrine state, follicular development, or ovulation	Closer biological reference standard; limited by sampling cost, delay, hygiene, and routine availability.	Calibrates labels and prevents acoustic behaviour from being equated with ovulation [16,36,37].
Computer vision/thermal imaging: posture, mounting, standing, movement, and temperature	Rich pen-level context; vulnerable to occlusion, lighting, contamination, identity tracking, and processing load.	Supports source confirmation and multimodal sow monitoring [24,79,87].
Acoustic monitoring: calling rate, call type, amplitude, duration, and spectral structure	Non-contact and continuous; limited by noise, overlapping calls, caller attribution, and indirect biological specificity.	Useful for early warning or verification; insufficient alone for ovulation timing [38,39,40,41,42,43].
Multimodal decision support: behavioural, physiological, identity, and management evidence	Reduces single-sensor failure; adds integration cost, synchronisation, missing data, and alert design challenges.	Most defensible route to actionable farm alerts [17,30,48,87,93].

Note: Modalities measure different points in the reproductive evidence chain and should not be treated as interchangeable ground truths.

## Data Availability

No new data were created or analysed in this study. Data sharing is not applicable to this article.
